# The impact of dislocations on AlGaN/GaN Schottky diodes and on gate failure of high electron mobility transistors

**DOI:** 10.1038/s41598-020-73977-2

**Published:** 2020-10-14

**Authors:** Sven Besendörfer, Elke Meissner, Farid Medjdoub, Joff Derluyn, Jochen Friedrich, Tobias Erlbacher

**Affiliations:** 1 Fraunhofer Institute for Integrated Systems and Device Technology IISB, Schottkystr. 10, 91058 Erlangen, Germany; 2 Chair of Electron Devices (LEB), University Erlangen-Nürnberg, Cauerstr. 6, 91058 Erlangen, Germany; 3 CNRS-IEMN, Institute of Electronics, Microelectronics and Nanotechnology, Avenue Poincaré, 59650 Villeneuve d’Ascq, France; 4 EpiGaN, Kempische Steenweg 293, 3500 Hasselt, Belgium

**Keywords:** Electronic devices, Characterization and analytical techniques

## Abstract

GaN epitaxially grown on Si is a material for power electronics that intrinsically shows a high density of dislocations. We show by Conductive Atomic Force Microscopy (C-AFM) and Defect Selective Etching that even for materials with similar total dislocation densities substantially different subsets of dislocations with screw component act as current leakage paths within the AlGaN barrier under forward bias. Potential reasons are discussed and it will be directly shown by an innovative experiment that current voltage forward characteristics of AlGaN/GaN Schottky diodes shift to lower absolute voltages when such dislocations are present within the device. A local lowering of the Schottky barrier height around conductive dislocations is identified and impurity segregation is assumed as responsible root cause. While dislocation related leakage current under low reverse bias could not be resolved, breakdown of AlGaN/GaN Schottky diodes under high reverse bias correlates well with observed conductive dislocations as measured by C-AFM. If such dislocations are located near the drain side of the gate edge, failure of the gate in terms of breakdown or formation of percolation paths is observed for AlGaN/GaN high electron mobility transistors.

## Introduction

The energy efficiency of applications like power converters and of the respective electronic devices is one of the essential points when dealing with a worldwide high demand on electrical energy. A strongly emerging material as the basis for such devices is GaN, which is commonly grown on SiC for high frequency or on Si for power electronic applications^1,2^. However, cost-effective heteroepitaxial growth of GaN goes along with high defect densities as compared to a pure GaN based approach. Clarifying the role of defects in terms of their impact on device performance and reliability needs to fulfill several requirements when aiming for an overall technology improvement: the ability to detect defects, the development of an understanding of how they form and what consequences they have on local material properties as well as the relation of this knowledge to the behavior of devices. Especially the last point is very difficult to accomplish from an experimental point of view when high densities of defects are involved and many of them are contained within each device. Consequently, pure device-based studies are always speculative to some extent when concluding specific types of defects as origin of observed abnormalities. Similarly, pure material-based investigations can only guess that observed defects have a detectable influence on a device. The desired approach should always be a parallelized material and device characterization. Having materials that to one’s best knowledge differ in the density of one specific type of defect only may allow conclusions by comparing each material’s device data. In case of GaN on Si, this was successfully demonstrated when V-pits were proven as crucial for vertical breakdown^3,4^. However, in the case of threading dislocations (TDs), the situation becomes more complex. There are different types of dislocations and their electrical properties are not only dependent on the type, but also on the growth conditions used, i.e. they are characteristic for the specific sample under investigation^5–7^. Thus, comparing samples and corresponding devices only in terms of the materials’ total dislocation densities or even the densities of single dislocation types is not a suitable approach. Indeed, the dislocations’ electrical properties need to be analyzed for each sample explicitly, which requires a very thorough and complete material characterization^8^. Therefore, it is not surprising that studies report a large diversity in results^5–20^. Some elucidate that mainly threading screw dislocations (TSDs) are the main cause of leakage currents^9–12^. Others find that also threading mixed type dislocations (TMDs) play a critical role or that neither of both types seem to show an influence on device characteristics^7,8,13^. Also, subsets of certain dislocation types have been observed to be potentially critical or that dislocations even can act as centers where current is blocked^8,12,14^. Total dislocation densities in GaN on Si are in the order of $10^{9} {\text{ cm}}^{ - 2}$, i.e. devices typically cover a large amount of dislocations. Consequently, it is not clear whether single dislocations have an influence on a device or rather their entirety or local arrangement. Thus, a direct correlation between dislocations and devices is needed, which of course requires the characterization of very small devices in combination with a mature material investigation within a sophisticated design of experiment. There are essentially two ways to do so: (1) investigating the material underneath a device after its electrical characterization or (2) fabricating devices on a pre-characterized area. The first way might be easier to perform as conspicuous devices can be identified first and selective material analysis can be carried out. However, processing steps such as annealing or metallization or device failure itself can irreversibly change the underlying material and the chances for a successful reverse-engineering on the properties of intrinsically grown-in material defects are low, especially for surface-near defects. Still, for GaN on GaN this approach allowed the direct identification of TDs as being responsible for leakage current in p-n-diodes under high reverse bias^21^. The second approach allows characterization of the as-grown material first. However, it is challenging to fabricate devices on a microstructurally pre-characterized area, especially when small devices of only a few µm^2^ are needed for probing single dislocations. Moreover, one has to ensure that material characterization does not change the material or its surface to such extent that the behavior of the device is dominated by this^16,19^. In any case, as good statistics as possible are essential. There are non-controllable effects like singular processing issues or spatially non-detectable defects like point defects and their complexes that may cause severe fluctuations of the microscopic devices’ behavior. However, if a statistically substantial relation between a specific device characteristic and certain dislocations is found, it is a direct proof. To our knowledge, in terms of dislocations there is no study for GaN on Si, which goes such direct way and succeeds in directly observing the dislocations’ impact on devices.

In this work we investigate two AlGaN/GaN high electron mobility transistor (HEMT) structures with focus on the very near-surface AlGaN barrier layer. Its defect content in terms of TDs and their electrical properties may be crucial for transistor performance and reliability aspects, such as gate leakage or gate breakdown. First, we perform an in-depth material analysis in order to reveal potential differences in AlGaN barrier TD densities, types, electrical properties, morphology and composition. We present the methodology for exclusively measuring the electrical properties of TDs in the barrier layer. Then, we investigate the influence of certain single dislocations on the current–voltage forward characteristics of micrometer sized AlGaN/GaN Schottky diodes in a direct way and explain the observations in a model of parallel combination. The experimental realization of this approach is  explained in detail. The influence of certain dislocations under high reverse bias is shown for HEMTs in off-state in terms of gate failure and for Schottky diodes in terms of breakdown in a relative comparison of both samples.

## Results and discussion

### Methodology for investigating the AlGaN barrier in terms of leakage current paths

**Figure 1:**
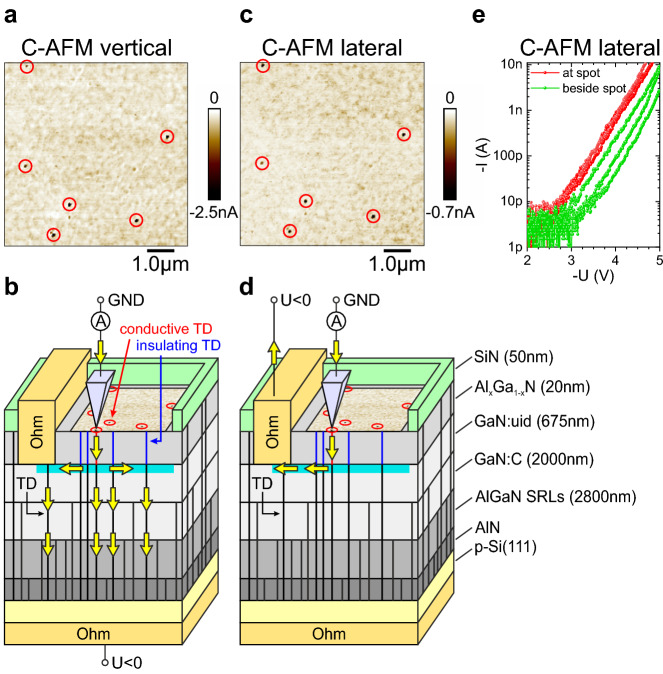
Correlation of vertical and lateral C-AFM measurements on an identical area of the sample measured in forward bias direction. (**a**) Vertical C-AFM measurement at a forward bias of -4.5 V showing leakage spots marked by red circles. (**b**) Corresponding vertical setup and predicted current flow. (**c**) A Lateral C-AFM measurement at a forward bias of − 4.5 V recorded directly after the vertical measurement shows leakage spots at identical positions as observed vertically. The current values are lower compared to a measurement on a fresh location due to surface oxidation during the vertical measurement. (**d**) Corresponding lateral setup and current flow. (**e**) Current voltage characteristics at the positions of leakage spots and beside as measured in lateral C-AFM setup.

In the following we investigate two samples A and B. They exhibit an identical layer stack grown on Si(111) for AlGaN/GaN HEMTs. Figure 1d shows the structure of both samples schematically. The only difference between both samples is the growth temperature of the AlN nucleation layer. We followed the study by Freedsman et al.^22^ and optimized the nucleation conditions for sample B in terms of V-pit formation. The intention was to provoke different microstructural properties in all layers above the nucleation layer up to the AlGaN barrier^23,24^. As we want to focus on dislocations in the AlGaN barriers of both samples, we need to find a way to characterize them electrically. By vertical conductive Atomic Force Microscopy (C-AFM) measurements it was shown in the past that dislocations meeting the sample surface of an AlGaN/GaN heterostructure are centers of vertical leakage current^8^. Although it is not clear how the leakage current explicitly flows through the layer structure in vertical direction and no direct proof was shown, it could be concluded that the two dimensional electron gas (2DEG) at the AlGaN/GaN interface plays an essential role for vertical leakage as it realizes a parallel combination of underlying electrically conductive defects^8^. Thus, spatial differences in the extent of vertical leakage current measured in such vertical C-AFM setup should mainly have their origin in the AlGaN barrier layer directly above the 2DEG and therefore should allow conclusions about the electrical properties of dislocations within the AlGaN barrier. In Fig. 1a such a vertical C-AFM measurement is exemplarily shown for sample A, where the AFM-tip was grounded and a negative bias of − 4.5 V was applied to the Si substrate. In the measured region, the SiN capping layer was removed. The setup and predicted current flow is schematically depicted in Fig. 1b. Discrete points of high vertical leakage current are observed analogous to previous studies^6,8–12,16,17,19^. Figure 1c shows the same area, but measured in a lateral configuration after finishing the vertical measurement. The bias, which was applied to an Ohmic contact to the 2DEG instead, was − 4.5 V as well. This setup can be considered as an AlGaN/GaN Schottky diode with a scanning Schottky contact, where a negative bias results in a negative current and corresponds to the diode’s forward direction in our case. Switching between vertical and lateral configuration was carried out during scanning. It can be seen that indeed the distribution of leakage spots is identical, which also means that no additional relevant defects in terms of producing leakage current were induced by electrical  stressing within the first C-AFM measurement. However, in the lateral C-AFM configuration as shown in Fig. 1d the explicit current flow is known and spatial differences are given by spatially different electrical properties of the AlGaN barrier. This means the vertical setup in Fig. 1b is suitable to characterize local differences in the electrical properties of the AlGaN barrier, but without any Ohmic contact processing on the sample surface being necessary. Current–voltage (I(V)) characteristics measured laterally on leakage spots and beside are presented in Fig. 1e. An exponential behaviour with similar exponent is observed in both cases. However, the current level at leakage spots is reproducibly higher. The I(V) curves measured at different leakage spots are very similar and tend to vary more for locations between the leakage spots. This seems indicative for a defect with characteristic electrical properties as origin of the leakage spots and a spatially inhomogeneous material in terms of electrical properties in between. However, no large amount of measurements was carried out and thus this conclusion is speculative at this point. Care has to be taken, when interpreting the absolute values of the current as surface oxidation during C-AFM measurements and especially during  recording of I(V) curves is a severe issue^16,19^. Consequently, also  the current values observed in Fig. 1c are expected to be substantially lower than expected for a measurement on a fresh region. No current above noise level could be detected in reverse direction for both C-AFM configurations.

### Leakage current and relation to dislocation types

**Figure 2:**
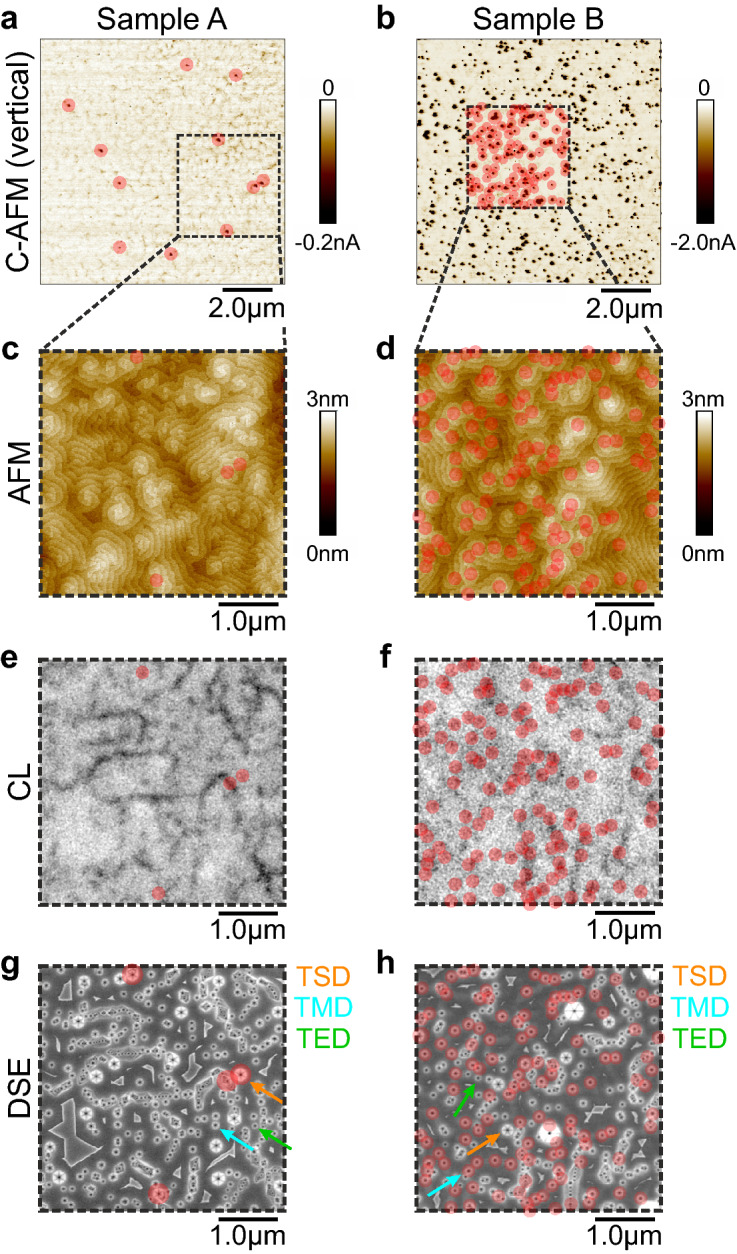
Direct correlational material investigation of both samples under forward bias. In (**a**) and (**b**) vertical C-AFM mappings of sample A and B respectively show leakage current paths through the AlGaN barrier as exemplarily indicated by red circles. A bias of − 3 V was used in (**a**) and of − 3.5 V in (**b**). In (**c**) and (**d**) topography images of the areas marked by dashed squares in (**a**) and (**b**) as measured by tapping AFM are depicted. The red circles are the overlayed positions of the leakage current paths. Corresponding panchromatic CL mappings in (**e**) and (**f**) reveal the distribution of non-radiative defect recombination centers. They match with the distribution of dislocations as can be observed by etch pits after DSE in (**g**) and (**h**). An overlay of the leakage current paths exhibits a subset of TSDs as conductive for sample A and a subset of TMDs for sample B as identified by the etch pit diameters (large = TSD, medium = TMD, small = TED).

As mentioned, defect and especially dislocation related leakage can vary significantly between individual samples. Thus, both samples were investigated in detail with regard to the electrical and morphological properties of the AlGaN barrier. Following findings above, electrical characterization of the barrier was accomplished by vertical C-AFM measurements. The results after applying the methodology as described elsewhere^8^ are summarized in Fig. 2. A bias of − 3 V was used in Fig. 2a and of − 3.5 V in Fig. 2b. As observed, the density of leakage paths through the AlGaN barrier is substantially different between both samples. Sample A exhibits a density of approx. $2 \times 10^{7} {\text{ cm}}^{ - 2}$, sample B of approx. $3 \times 10^{8} {\text{ cm}}^{ - 2}$. The corresponding topographies of the AlGaN layers at the same places are shown in Fig. 2c,d as measured by AFM in tapping mode. For both samples, leakage paths correlate well with hillocks, which usually form around screw or mixed type dislocations. However, sample A exhibits many hillocks that contain no leakage path which is in strong contrast to sample B, where most of all hillocks give rise to leakage current. All underlying TDs can be seen in Fig. 2e,f by means of dark spots in panchromatic Cathodoluminescence (CL) measurements and of etch pits after defect selective etching (DSE) in Fig. 2g,h. Sample B exhibits only a slightly lower TD density of $2.1 \times 10^{9} {\text{ cm}}^{ - 2}$, as compared to $2.8 \times 10^{9} {\text{ cm}}^{ - 2}$ of sample A, which underlines that the total TD density is not the origin for the much higher leakage spot density in sample B. Here, the TD densities were quantified by manually counting the etch pits observed on a scale of $20 \times 20\:\upmu {\text{m}}^{2}$ by Scanning Electron Microscopy (SEM). Such scale contains around $10^{4}$ etch pits and is considered to be representative for the whole sample. A thorough analysis of the etch result reveals that both samples exhibit an identical TSD density of $6.9 \times 10^{7} {\text{ cm}}^{ - 2}$. A reliable discrimination between TMDs and threading edge type dislocations (TEDs) is not always possible. However, a chain-like arrangement of TDs is preferably observed in sample A and indicative for low-angle grain boundaries decorated by TEDs. Thus, a similar TMD density, but higher TED density for sample A can be assumed. A direct correlation between the C-AFM and DSE results shows that nearly all AlGaN barrier leakage paths in sample A correspond to TSDs, but that there are many TSDs that do not serve as leakage path at the same time. As the TSD density is approx. $6.9 \times 10^{7} {\text{ cm}}^{ - 2}$, this means that only around 30% of them produce leakage. The situation for sample B is completely different. Nearly no leakage spots occur at TSDs, but correlate with smaller etch pits that are assigned to TMDs after verifying the presence of a screw component with the help of the topography image. However, there is a portion of TMDs showing no leakage. In order to gain more insight into the potential mechanism behind, the AlGaN barrier morphologies were investigated.

### AlGaN barrier morphology and relation to conductive dislocations

**Figure 3:**
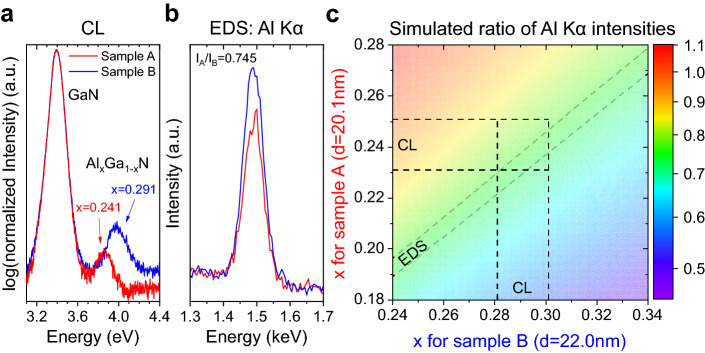
CL and EDS characterization of the AlGaN/GaN heterostructure. (**a**) CL spectra recorded on-plane for both samples show a significant shift of the AlGaN barrier NBE emission peak position to higher energy for sample B. This corresponds to a difference of 0.05 in x. EDS measurements of the Al Kα intensities in (**b**) verify this result in combination with the simulation shown in (**c**) and exclude a difference in the NBE emission peak position dominantly induced by strain.

The root-mean-squared (rms) roughness on a scale of $20 \times 20\:\upmu {\text{m}}^{2}$ is very similar for both samples: 0.8 nm for sample A and 1.0 nm for sample B. X-Ray Reflectometry (XRR) measurements show that the difference in barrier thickness is around 10%. A value of 20.1 nm is obtained for sample A and  of 22.0 nm for sample B. The Al-content was first investigated via the position of the near band edge (NBE) emission peak measured by spectroscopic CL as shown in Fig. 3a. The Al mole-fraction x within Al_x_Ga_1-x_N was calculated using the relation $E_{{\text{g}}} \left( x \right) = xE_{{\text{g}}}^{{{\text{AlN}}}} + \left( {1 - x} \right)E_{{\text{g}}}^{{{\text{GaN}}}} - bx\left( {1 - x} \right)$, where $E_{{\text{g}}} \left( x \right)$ was assumed to be identical with the fitted NBE emission peak position^25^. The bandgaps of AlN and GaN at room temperature were assumed to be $E_{{\text{g}}}^{{{\text{AlN}}}} = 6.13 {\text{ eV}}$^25^ and $E_{{\text{g}}}^{{{\text{GaN}}}} = 3.39 {\text{ eV}}$^26,27^. For the bowing parameter a value of $b = 1.0$ was taken according to former studies^26^. This results in x = 0.241 for sample A and x = 0.291 for sample B with an estimated error of ${\Delta }x = 0.01$. As the position of the NBE emission peak is also sensitive to strain it is not clear whether the observed positions are due to different Al-contents only. Thus, we carried out on-plane EDS measurements on both samples under identical conditions after local removal of the SiN capping layer. We observe a ratio of $I_{{{\text{Al}} {{\text{ K}\alpha }}}}^{{\left( {\text{A}} \right)}} /I_{{{\text{Al}} {{\text{ K}\alpha }}}}^{{\left( {\text{B}} \right)}} = 0.745$, where $I_{{{\text{Al}} {{\text{ K}\alpha }}}}^{{\left( {\text{A}} \right)}}$ and $I_{{{\text{Al}} {{\text{ K}\alpha }}}}^{{\left( {\text{B}} \right)}}$ are the intensities of the Al Kα lines of sample A and B respectively like shown in Fig. 3b. This experimental value was compared to simulated ones for different x for both samples around the x-values obtained by CL. The barrier thicknesses were kept constant at the values measured by XRR. The result is illustrated using Fig. 3c. The set of values for the ratio $I_{{{\text{Al}} {{\text{ K}\alpha }}}}^{{\left( {\text{A}} \right)}} /I_{{{\text{Al}} {{\text{ K}\alpha }}}}^{{\left( {\text{B}} \right)}}$ predicted by the simulation for the x-values obtained by CL intersects with the contour line corresponding to the value measured by EDS. This shows that strain has a negligible influence on the NBE emission peak positions in our case and the AlGaN-barriers indeed have a substantially different Al-content, which is therefore  the only resolved morphological difference between both AlGaN barriers.

A general unintended shift of the process parameters can be excluded as the Al-contents of the AlGaN strain-relief layers are identical for both samples as verified by cross-sectional EDS. Therefore, we assume a morphological root cause. One potential mechanism is the Al pulling effect similar to the In pulling effect in InGaN layers grown on GaN^28–30^. Different nucleation conditions as used in our work were shown to induce different depth profiles of the grown-in V-pit density, which in turn has an influence on stress relaxation and thus on Al incorporation^3,24,30,31^.

We can only speculate about the origin for enhanced leakage observed at TSDs and TMDs within the AlGaN barrier as well as the difference between both samples. Al was observed to segregate at dislocation cores in AlGaN forming Al depletion zones around and potentially influencing the electrical properties^32–34^. However, this effect was consistently reported to be most prominent at TEDs. On the other hand, Al shows a high reactivity with oxygen leading to a promoted oxygen incorporation in AlGaN layers of higher Al content^35,36^. Oxygen has been shown to segregate at or around dislocation cores resulting in the formation of shallow donor trap states and causing that not only the dislocation type itself is decisive for the electrical properties, but the explicit atomic core configuration^11,37–42^. A region of high n-doping around TDs, which is several times larger than the structural dimension of the defect itself was shown by combined C-AFM and Scanning Capacitance Microscopy (SCM) measurements in the past^43^. Recently, a direct evidence for the decoration of certain TSDs and TMDs by oxygen was shown^44^. One possible explanation was attributed to the fact that there are closed and open core configurations within each type of TDs dependent on the presence of dopants and impurities^45^. More work is needed on our samples in order to clarify the explicit mechanism. However, it is shown that morphological differences in terms of defects and strain within the buffer layers can cause fundamentally different electrical properties of the AlGaN barrier layers despite being grown under same conditions and exhibiting nearly identical TD densities.

### Direct correlative experiment for elucidating the impact of single conductive dislocations on diodes

**Figure 4:**
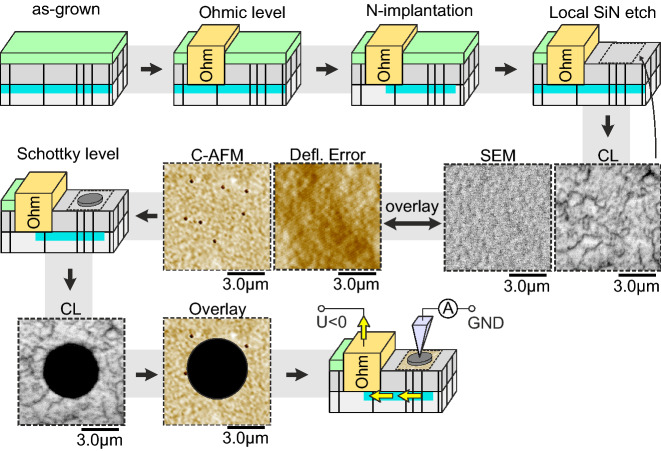
Workflow for the setup of a direct correlative experiment in order to elucidate the influence of single conductive dislocations on micro diodes. The C-AFM measurement in this scheme was carried out at bias of − 7 V, where black color corresponds to − 13 nA and below and white color to − 1 nA and above.

For both samples, the observed leakage current at TDs in C-AFM scans is around two orders of magnitude higher as compared to surrounding areas. The lateral extent of a TD’s conductive region is assumed to be equal to the diameter of the observed leakage spots, which is typically around 100–300 nm and thus larger than the lateral resolution in C-AFM scans. A doubling of the forward current is expected for a circular AlGaN/GaN Schottky diode of 3 µm in diameter under the presence of one such conductive TD. As sample A shows a much lower density of conductive dislocations it is predestined for a direct correlational approach. An active 2DEG with an area of $1 \times 1 {\text{ mm}}^{2}$ was prepared by isolation towards the sample edges through N implantation. One edge of the 2DEG was contacted by a large Ohmic contact and the SiN capping layer was locally removed from the 2DEG area. This is schematically presented in Fig. 4. With the help of small Ohmic contacts as orientation markers a direct correlative material investigation by C-AFM and CL was carried out, following the procedure described elsewhere^8^. Subsequently, the Schottky level was processed and the explicit positioning of Schottky pads with diameters of 3 µm and 5 µm on the pre-characterized area was investigated by mapping CL again and overlaying on the initial results. This finally allows an overlay of the Schottky pads on the C-AFM images. Consequently, a direct correlation of the current-density-voltage (J(V)) characteristics recorded between such Schottky pads and the large Ohmic contact in forward direction with underlying conductive dislocations in forward direction as measured by C-AFM is enabled.

### Forward characteristic of micro diodes and relation to single conductive dislocations

**Figure 5:**
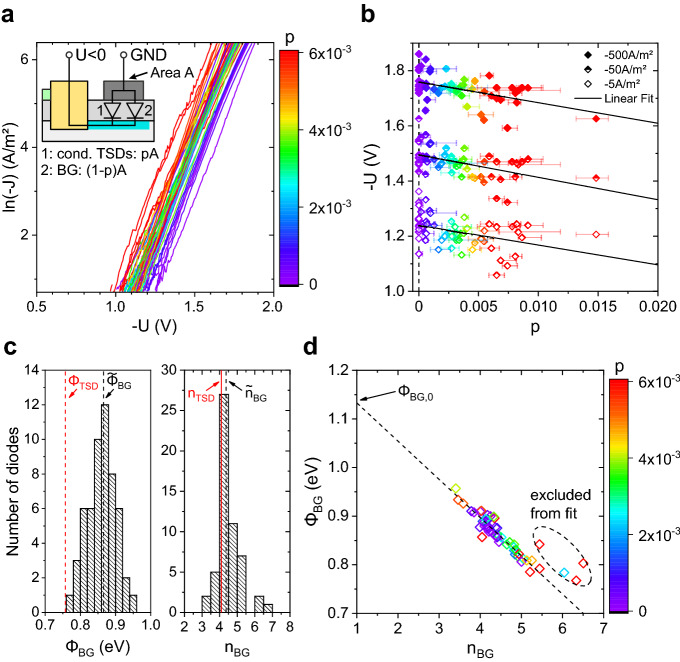
Results from the direct correlative experiments with micro diodes. (**a**) Current-density-voltage characteristics of all measured diodes in forward direction clearly show a shifting to lower absolute values of the voltage for increasing values of *p*, which is the areal fraction of conductive dislocations underneath the Schottky contact. This is quantitatively illustrated in (**b**) and modelled by a parallel combination of two diodes within the frame of thermionic emission. Diode 1 represents the entirety of conductive dislocations underlying the micro diode, Diode 2 the remaining area respectively. In (**c**) the fit result is shown, where the dashed black lines indicate the median values of the histograms. A linear relation between the apparent background Schottky barrier heights and the corresponding ideality factors is observed in (**d**).

In Fig. 5a, all obtained J(V) curves from sample A are depicted, where the area $A^{\left( i \right)}$ of diode $i$ was explicitly measured by SEM for all $i$. Substantial differences in the current levels were observed. A common approach to describe diodes fabricated on a material with patches of different electrical properties is the parallel combination model^46^. In our case, as we deal with sample A only, the patches are the conductive TSDs embedded in an inhomogeneous background. In order to correlate the measured J(V) curves of all micro diodes with their corresponding content of conductive TSDs, the patches’ portion $p^{\left( i \right)}$ of each micro diode’s area $A^{\left( i \right)}$ is needed. This requires a systematic and reliable marking of conductive TSDs within C-AFM measurements. The procedure for extracting all $p^{\left( i \right)}$’s is described in the supplementary material. The result is illustrated in Fig. 5a by the color-coding and quantitatively in Fig. 5b. In Fig. 5a it can be clearly observed that for constant $\text{J}$ the J(V) curves shift to lower values of − U with increasing $p$, which is consistent to a decreasing turn on voltage. This decrease of –U with increasing $p$ for several fixed current densities is presented in Fig. 5b. From Fig. 5a it is evident that diodes even with very similar values for $p$ exhibit small variations in slope, but larger ones in intercept. This especially also holds true for diodes with $p = 0$. However, according to the fact that all spots correspond to certain TSDs we assume all of them to be identical in their electrical properties. In a model of parallel combination assuming thermionic emission and following sign convention of the C-AFM setup we get the following sum of two diodes for each micro diode $i$, one corresponding to the inhomogeneous background area and one to conductive TSDs:

$$J^{\left( i \right)} \left( V \right) = - A^{*} T^{2} \left[ {\left( {1 - p^{\left( i \right)} } \right)\exp \left( { - \beta \phi_{{{\text{BG}}}}^{\left( i \right)} } \right)\exp \left( { - \frac{\beta V}{{n_{{{\text{BG}}}}^{\left( i \right)} }}} \right) + p^{\left( i \right)} \exp \left( { - \beta \phi_{{{\text{TSD}}}} } \right)\exp \left( { - \frac{\beta V}{{n_{{{\text{TSD}}}} }}} \right)} \right]$$

Here, $T = 300 {\text{ K}}$ is the ambient temperature and $\beta = e/k_{{\text{B}}} T$ with $e$ as elementary charge and $k_{{\text{B}}}$ as Boltzmann constant. $\phi_{{{\text{BG}}}}^{\left( i \right)}$ and $n_{{{\text{BG}}}}^{\left( i \right)}$ are the apparent Schottky barrier height and ideality factor of the background diode $i$ and $\phi_{{{\text{TSD}}}}$ and $n_{{{\text{TSD}}}}$ the apparent Schottky barrier height and ideality factor of the diode corresponding to conductive TSDs. A fit of this model was performed simultaneously to all measured J(V) curves by using $\phi_{{{\text{BG}}}}^{\left( i \right)}$ and $n_{{{\text{BG}}}}^{\left( i \right)}$ as micro diode specific free parameters and $\phi_{{{\text{TSD}}}}$ and $n_{{{\text{TSD}}}}$ as globally shared free parameters. The fixed values for $p^{\left( i \right)}$ were taken from C-AFM  measurements and the effective Richardson constant $A^{*}$ was linearly interpolated using $x = 0.241$ according to CL as follows^47^:

$$A^{*} \left( x \right) \approx \frac{{4\pi ek_{B}^{2} }}{{h^{3} }}\left[ {xm_{{{\text{AlN}}}}^{*} + \left( {1 - x} \right)m_{{{\text{GaN}}}}^{*} } \right]$$

$h$ is the Planck constant, $m_{{{\text{AlN}}}}^{*} = 0.35m_{0}$^48^ and $m_{{{\text{GaN}}}}^{*} = 0.22m_{0}$^49^ with $m_{0}$ as the electron’s rest mass. The obtained individual values for $\phi_{{{\text{BG}}}}^{\left( i \right)}$ and $n_{{{\text{BG}}}}^{\left( i \right)}$ show substantial variation as depicted by the histograms in Fig. 5c. However, $\phi_{{{\text{BG}}}}^{\left( i \right)}$ and $n_{{{\text{BG}}}}^{\left( i \right)}$ are dependent. In Fig. 5d a linear decrease of $\phi_{{{\text{BG}}}}^{\left( i \right)}$ with increasing $n_{{{\text{BG}}}}^{\left( i \right)}$ is observed. Such behaviour was already observed in the past^43,50,51^ and is usually accorded to local deviations of the barrier height from the value characteristic of uniform interfaces^46,52^. If we compare the background areas of different micro diodes in terms of the local deviations of the measured current in C-AFM mappings, we cannot observe any substantial differences between different diodes. Thus, we do not think intrinsic local deviations of the barrier height are likely. Effects that are more prominent in stationary J(V) measurements than in scanning measurements pose more likely reasons instead. Charge carrier trapping by defects as for instance vastly present non-conductive TDs, as observed in Fig. 2e,g, is one example. Another reason might be an inhomogeneous surface oxidation by prior C-AFM measurements. Still, the extrapolated homogenous Schottky barrier height of the background area is $\phi_{{{\text{BG}},0}} = 1.13 {\text{ eV}}$, which is in good agreement to formerly published values for Al_0.25_Ga_0.75_N/GaN diodes based on Ni/Au Schottky contacts^50^. Despite allowing individual apparent Schottky barrier heights for the background areas, the assumed model gives a Schottky barrier height at the position of conductive TSDs, which is consistently lower. This shows that there are indeed two simultaneous effects that determine the J(V) characteristics: the local presence of conductive TSDs and an overall inhomogeneity with not precisely known origin. Taking the median value $\tilde{\phi }_{{{\text{BG}}}}$ of all measured $\phi_{{{\text{BG}}}}^{\left( i \right)}$’s as the typical apparent barrier height of the background area, the typical barrier lowering at conductive TSDs is $0.1 {\text{ eV}}$ as registered by the device. A Schottky barrier lowering at dislocations was already reported in the past. In the presence of dislocations with a screw component, it was assigned to coexisting donor-like trap states in their vicinity^20,53^. As discussed, a potential origin of local n-doping in AlGaN-barriers is a locally increased oxygen incorporation as for example due to its segregation at dislocation sites^11,37–42^. At the same time, $n_{{{\text{TSD}}}}$ practically equals the median value of all measured $n_{{{\text{BG}}}}^{\left( i \right)}$’s. This results in an expected typical difference in background and TSD current of around factor 50, which is in good agreement with results obtained by C-AFM mappings. Consequently, the influence of single conductive TDs on suitably small diodes can be confirmed.

### Dislocations and their impact on diodes’ reverse characteristics and gate failure of AlGaN/GaN HEMTs

In former studies it was vastly reported about dislocations serving as leakage current paths in reverse direction^6,9–11,16,17,19^. As mentioned, we could not resolve any reverse current by C-AFM measurements, which made a direct correlative approach as carried out for forward direction impossible. However, the C-AFM setup was limited to a reverse bias of 10 V. In the past, it was shown that the observation of TSDs and TMDs as leakage current paths in reverse direction is strongly dependent on applied reverse bias^16^. In other words, dislocations that occur as conductive in forward direction in our case might also produce significant leakage in reverse direction when applying biases greater than + 10 V. As I(V) recording for high reverse biases needed a different setup than C-AFM, which in turn was limited to much larger devices, only a relative comparison of sample A and B in terms of the reverse characteristic of macroscopic diodes was possible.

**Figure 6:**
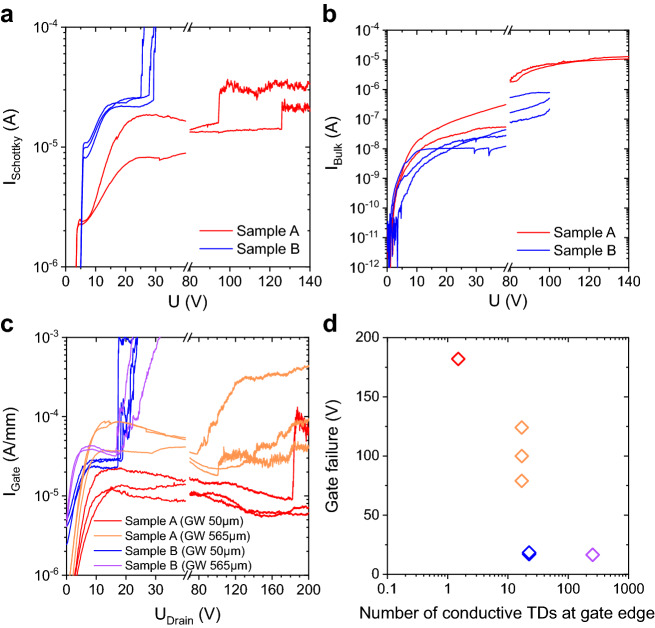
Results from reverse bias characterization of macro diodes and OFF-state measurements of HEMTs. (**a**) The measured current through diodes biased in reverse direction show an early breakdown for sample B as compared to sample A. This breakdown occurs within the AlGaN barrier as verified by simultaneously measured vertical leakage shown in (**b**). Equivalently gate leakage current in (**c**) is observed to increase rapidly at much lower values of drain bias for sample A than compared to B. In (**d**) the drain bias at which failure is observed is related to the number of critical dislocations located at the drain side of the gate edge as statistically expected from C-AFM measurements.

In Fig. 6a the reverse characteristics of several $100 \times 100\:\upmu {\text{m}}^{2}$ sized macroscopic diodes on each sample are shown. Schottky and backside contacts were grounded and a voltage sweep was applied to the Ohmic contact. It can be clearly observed that all diodes fabricated on sample B show a much earlier failure at around 20–30 V as compared to diodes on sample A showing failure typically in the range 90–130 V. The corresponding failure is irreversible and takes place within the AlGaN-barrier as verified by simultaneously measuring the vertical bulk leakage current shown in Fig. 6b. It is worth mentioning again, that sample B has the slightly lower TD density within the AlGaN barrier and an identical TSD and similar TMD density as compared to sample A. Despite that, the breakdown voltage is a factor of 5 lower. This shows again that neither the total TD density nor the density of single TD types is crucial a priori. Instead, it is likely that TDs which were observed as conductive in forward direction also play a critical role in reverse direction, but only under high electrical fields and thus the individual electrical properties of TDs are decisive.

Beside macroscopic Schottky diodes, normally-on AlGaN/GaN HEMTs with locally etched SiN beneath the gate contact and with gate widths of 50 µm and 565 µm on each sample were investigated. Channel length (28 µm), gate length (2 µm), gate to source spacing (4 µm) and drain to gate spacing (18 µm) were identical for all HEMTs. Source and backside contact were grounded, the gate voltage was set to 1 V below threshold voltage, which was $V_{{{\text{th}}}} = - 3.68\text{ V}$ for sample A and $V_{{{\text{th}}}} = - 5.85\text{ V}$ for sample B and a voltage sweep was applied to the drain. The measurement results are depicted in Fig. 6c. Equivalent to measurements on diodes, an early OFF-state breakdown due to failure of the AlGaN-barrier takes place for HEMTs fabricated on sample B, which is independent of gate width at around 20 V. However, sample A exhibits a failure of the AlGaN-barrier which seems dependent on gate width. For 50 µm, breakdowns of 182 V and greater than 200 V were observed, for 565 µm breakdown occurred between 79 and 124 V. Such dependency is indicative for a defect-driven process. It was reported that gate breakdown primarily appears at the gate edge towards drain due to local pit formation induced by high electric fields^54^. A relation between such pits and dislocations in general^55,56^ as well as dislocations with screw component was reported^57^. However, also the mobility of dislocations was observed to play an important role as inverse piezoelectric stressing due to high electric fields promotes dislocation movement towards the gate edge^58^. Reverse leakage through the AlGaN barrier can cause further damage along or near initial conductive paths like TDs leading to an increase in deep level density and the formation of percolation paths, which lead to a noisy behaviour of gate current and finally to irreversible failure in terms of breakdown^55,59,60^. Such noisy behaviour in gate current is observed in our case for sample A. If we assume that initial defects being critical for reverse direction are dislocations that were observed as conductive in forward direction by C-AFM, we can relate the appearance of gate current abnormalities to the statistically expected number of critical dislocations near the gate edge towards the drain. By abnormalities we mean either a steep increase of the gate current or the beginning of a noisy behaviour. The idea is that the probability for the formation of percolation paths should correlate with the amount of initially present critical defects, at least when TD movement can be neglected. The result is reproduced in Fig. 6d, where the critical area at the gate edge was calculated by the product of gate width with the typical diameter of dislocation spots as observed in Fig. 1a, which is approx. 150 nm. We observe a critical region for values between around 1 and 10 conductive TDs in forward direction.

In conclusion, we demonstrate the influence of conductive dislocations on forward and reverse characteristics of AlGaN/GaN Schottky diodes as well as on gate failure of AlGaN/GaN HEMTs in OFF-state. It is shown that a priori neither the total dislocation density nor the density of certain dislocation types is decisive, but their individual electrical properties that are given by the explicit atomic core configuration dominate. However, the appearance of specific core configurations and accompanying decoration by for example segregation of O at open cores seems dependent on epitaxial layer strain states and thus on nucleation conditions. In particular, TSDs and TMDs are observed to be critical in that manner and thus to selectively yield leakage current paths. The methodology for a material and device characterization in a direct correlational approach is presented. With this, a shift of the turn on voltage of AlGaN/GaN Schottky diodes to lower absolute values with an increasing density of conductive TDs within the AlGaN barrier underneath the Schottky contact is shown. By using a parallel combination model it turned out that the origin for that is a local lowering of the Schottky barrier height at such TDs. The same TDs that are observed to be highly conductive in forward direction did not show a detectable leakage current under low reverse bias, but are assumed to promote breakdown of AlGaN/GaN Schottky diodes and gate failure of AlGaN/GaN HEMTs in OFF-state when located at the drain side of the gate edge. Breakdown is assumed to occur according to the percolation theory.

## Methods

### Samples

Both samples were grown by Metal Organic Chemical Vapor Deposition (MOCVD) on p-type Si(111) of 6 inch in diameter and exhibit identical layer sequences and thicknesses. Following an AlN nucleation layer of around 200 nm in thickness, step-graded AlGaN strain relief layers were grown, consisting of 350 nm of Al_0.75_Ga_0.25_ N, 500 nm of Al_0.51_Ga_0.49_ N, 750 nm of Al_0.26_Ga_0.74_ N and 1200 nm of Al_0.13_Ga_0.87_ N (from bottom to top). An intrinsically C-doped GaN-layer of 2000 nm with a C-concentration of $3 \times 10^{19} {\text{ cm}}^{ - 3}$ as measured by Secondary Ion Mass Spectrometry (SIMS) and 675 nm unintentionally doped GaN serving as transistor channel layer followed on top. The samples’ surface layers are an Al_x_Ga_1-x_N barrier layer with a nominal value for x of 0.25 and for the thickness of 20 nm and an in-situ grown SiN capping layer with a thickness of 50 nm. Figure 1d schematically shows the structure under investigation. Growth conditions for all layers except the AlN nucleation layer were identical for both samples. The nucleation layer of sample B was optimized in terms of growth temperature compared to sample A by following the study of Freedsman et al.^22^.

### Material characterization

For AFM measurements in tapping mode as well as for C-AFM measurements a Bruker Dimension Icon was used (an introduction into the technique of C-AFM on AlGaN/GaN heterostructures can be found in^61^). For tapping mode highly doped Si-tips exhibiting a radius of curvature below 5 nm were used. C-AFM measurements were performed with tips with highly B-doped diamond-coating by using an extended tunnelling AFM (TUNA) module. These tips have a radius of curvature of 100–200 nm, but a nano-roughness in the 10 nm regime, which allows suitable spatial resolution. All measurements were carried out in air at room temperature. Panchromatic CL mappings were performed with an acceleration voltage of 5 kV, CL spectra were recorded with 4 kV using a $1200{\text{ mm}}^{ - 1}$ spectral grating. For CL measurements a Gatan MonoCL3 system with a photomultiplier and attached to a JEOL JSM-7500F SEM was used. The measurements were done under high vacuum (HV) conditions of $< 10^{ - 6}{\text{ mbar}}$ at room temperature. X-Ray reflectometry (XRR) was carried out by measuring the angle-dependent, specular reflection of a low angle incident Cu Kα1 beam on a Bruker Fabline diffractometer. Energy-dispersive X-Ray Spectroscopy (EDS) was carried out using an acceleration voltage of 3 kV and an X-Max Detector from Oxford Instruments attached to mentioned SEM. Supporting simulations were performed by the Monte-Carlo method. Defect selective etching was applied by etching the samples in KOH/NaOH eutectic melt at 450 °C for 4 min. A high-purity Ni sample holder for Si backside protection was used and samples were thereafter rinsed in DI-water and cleaned in a solution of concentrated sulphuric acid and hydrogen peroxide with a mixture ratio of H_2_SO_4_:H_2_O_2_ = 5:1 at 90 °C for 10 min.

### Device processing and direct correlation

Processing of devices consisted of four consecutive steps in total: (1) formation of Ti/Al/Ni/Au Ohmic contacts under annealing at 875 °C for 30 s, (2) isolation by N-implantation, (3) local etching of the SiN capping layer by dry etching and (iv) Ni/Au Schottky contact formation. The sequence of these steps is also shown in Fig. 4 for clarity. During annealing of Ohmic contacts, the surface was covered by the SiN capping layer in order to prevent relaxation of the AlGaN barrier during cool-down and thus the generation of additional defects^62^.

### Device characterization

The current–voltage characterization of the micro diodes was carried out by a two terminal configuration with the C-AFM setup described above, but by using PtIr-coated tips protruding from the end of the cantilever in order to enable a precise and directly visible positioning after suitable calibration. Electrical contacting of the small Schottky pads was done with the help of the AFM’s light microscopy and a calibrated contact force of 1 µN for all measurements. It was verified that this force is high enough to establish an optimal electrical contact, but low enough to not physically damage the microscopic Schottky pads. The corresponding large Ohmic contact was manually bonded, using a thin Au-wire and liquid silver paste. HEMT devices were measured with a Keithley SCS-4200 parameter analyzer.

## Supplementary information

Supplementary file 1

## Data Availability

The datasets generated during and/or analyzed during the current study are available from the corresponding author on reasonable request.
