# Supplementary material for: The impact of dislocations on AlGaN/GaN Schottky diodes and on gate failure of high electron mobility transistors
Source: Sci Rep. 2020 Oct 14;10:17252. doi: 10.1038/s41598-020-73977-2 (PMC7560755; doi:10.1038/s41598-020-73977-2)
Supplement: Supplementary file 1 [file 41598_2020_73977_MOESM1_ESM.pdf]

Supplementary material for:

The impact of dislocations on AlGaN/GaN Schottky diodes and on gate failure of high electron mobility transistors

S. Besendörfer[1,*], E. Meissner[1,2], F. Medjdoub[3], J. Derluyn[4], J. Friedrich[1] & T. Erlbacher[1,2]

[1]*Fraunhofer Institute for Integrated Systems and Device Technology IISB, Schottkystr. 10, 91058 Erlangen, Germany*

[2]*Chair of Electron Devices (LEB), University Erlangen-Nürnberg, Cauerstr. 6, 91058 Erlangen, Germany*

[3]*CNRS-IEMN, Institute of Electronics, Microelectronics and Nanotechnology, Avenue Poincaré, 59650 Villeneuve d'Ascq, France*

[4]*EpiGaN, Kempische Steenweg 293, 3500 Hasselt, Belgium*

**corresponding author: sven.besendoerfer@iisb.fraunhofer.de*

Calculation of the conductive dislocations' portion $p^{(i)}$ of each micro diode's area $A^{(i)}$. All fabricated micro diodes on sample A are located in a total of five C-AFM scans of at least $20 \times 20\ \mu\mathrm{m}^2$, containing several conductive TSDs. If $I^{(j)}_{\mathrm{C-AFM}}(x, y)$ is the negative (forward) current at location (x, y) and $A^{(j)}_{\mathrm{C-AFM}}$ the area of C-AFM scan j, a steep increase of the function

$$p^{(j)}_{\mathrm{C-AFM}}(I_0) := \frac{1}{A^{(j)}_{\mathrm{C-AFM}}} \iint\limits_{A^{(j)}_{\mathrm{C-AFM}}} \Theta^{(j)}(x, y; I_0)\, \mathrm{d}x\, \mathrm{d}y$$

with

$$\Theta^{(j)}(x, y; I_0) = \begin{cases} 1 \text{ for } I^{(j)}_{\mathrm{C-AFM}}(x, y) < I_0 \\ 0 \text{ else} \end{cases}$$

is expected, when $I_0 = I^{(j)}_{\mathrm{th}}$ equals the lowest background current levels. Thus, $I^{(j)}_{\mathrm{th}}$ is a good threshold value for discriminating between conductive TSDs and background in C-AFM scan j. In Supplementary Fig. 1a a representative C-AFM scan and in Supplementary Fig. 1b the corresponding function $p^{(j)}_{\mathrm{C-AFM}}(I_0)$ with its derivative $\partial \log p^{(j)}_{\mathrm{C-AFM}} / \partial I_0$ is shown. With this procedure, a distinct value $I^{(i)}_{\mathrm{th}}$ can be assigned to each diode

i, where $I_{\mathrm{th}}^{(i)} \equiv I_{\mathrm{th}}^{(j)}$ for all diodes in a common C-AFM image, because there was no detectable current gradient. Then, the following expression was applied to all diodes, where $A^{(i)}$ was explicitly measured by SEM for all i:

$$p^{(i)} = \frac{1}{A^{(i)}} \iint_{A^{(i)}} \Theta^{(j)}\left(x, y; I_{\mathrm{th}}^{(i)}\right) \mathrm{d}x\, \mathrm{d}y$$

Assymetrical error bars $\pm\Delta p^{(i)}$ were calculated according to the following expression by graphically estimating a symmetrical error $\Delta I_{\mathrm{th}}^{(i)}$ of $I_{\mathrm{th}}^{(i)}$:

$$\pm\Delta p^{(i)} = \frac{1}{A^{(i)}} \iint_{A^{(i)}} \Theta^{(j)}\left(x, y; I_{\mathrm{th}}^{(i)} \pm \Delta I_{\mathrm{th}}^{(i)}\right) \mathrm{d}x\, \mathrm{d}y$$

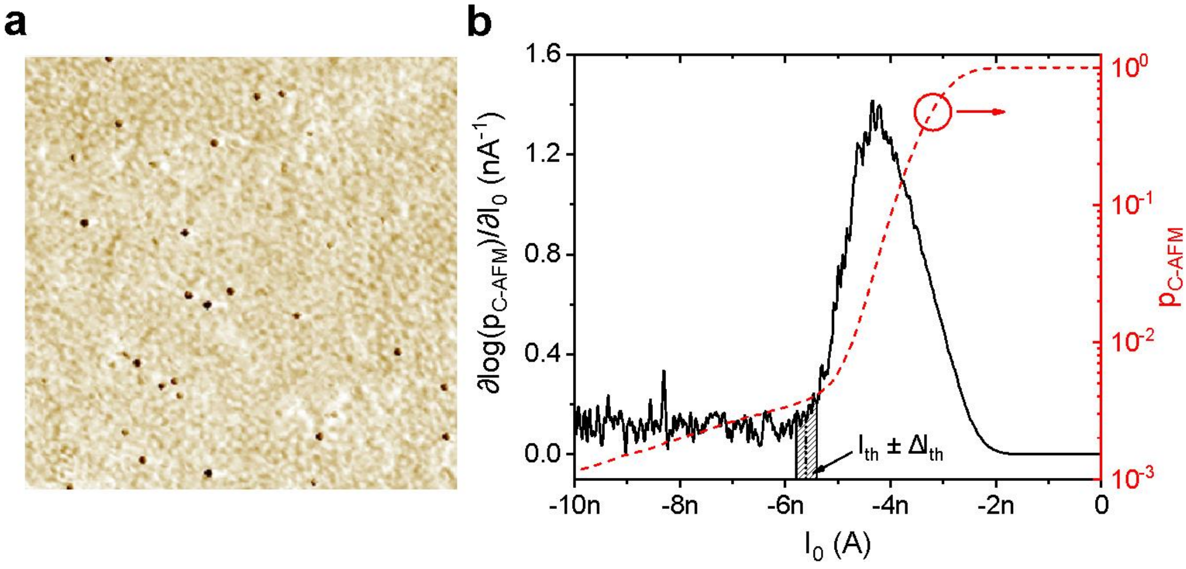

Supplementary Figure 1. Systematic determination of the threshold value I_{th}, which allows discriminating between conductive TDs and surrounding background. In (a) a typical C-AFM mapping is shown and in (b) the plot of the corresponding function $p_{\mathrm{C-AFM}}(I_0)$ is illustrated. The value of I_{th} is the position of the beginning steep increase of $p_{\mathrm{C-AFM}}(I_0)$, which can be systematically determined with the help of the derivative $\partial \log p_{\mathrm{C-AFM}}^{(j)} / \partial I_0$.
